# Review of Validated Methods to Evaluate Diet History in Diet Therapy and Counselling: An Overview and Analysis of Screeners Based on Food-Based Dietary Guidelines

**DOI:** 10.3390/nu15214654

**Published:** 2023-11-02

**Authors:** Laura Hoffmann, Sarah Egert, Joachim Allgaier, Kathrin Kohlenberg-Müller

**Affiliations:** 1 Department of Nutritional, Food and Consumer Sciences, Fulda University of Applied Sciences, 36037 Fulda, Germany; joachim.allgaier@oe.hs-fulda.de (J.A.); kathrin.kohlenberg-mueller@oe.hs-fulda.de (K.K.-M.); 2 Institute of Nutritional and Food Science, Nutritional Physiology, University of Bonn, 53115 Bonn, Germany; s.egert@uni-bonn.de

**Keywords:** diet quality, nutritional assessment, screener, screening tool, diet quality index, diet quality score, dietetic counselling, dietetic therapy

## Abstract

Evidence-based dietetic practice calls for systematically developed assessment methods for nutritional assessment in dietetic counselling and therapy (DCT). Screeners can provide a quick and easy way to determine a client’s diet quality and contribute to quality assurance in DCT. The aim of this systematic review was to give a comparative overview of screeners based on national food-based dietary guidelines (FBDGs) and to derive recommendations for developing an FBDG-based screener for DCT. The literature search in PubMed (MEDLINE), embase and Web of Science was conducted between May and July 2022, and updated in March 2023, in accordance with the consensus-based standards for the selection of health measurement instruments (COSMIN). The analysis focused on characteristics of screener design and measurement properties for screener testing. In total, 13 studies on 11 screeners based on FBDGs were included; 7 screeners were targeted to DCT. The content and scoring of screeners were based on the corresponding national FBDGs. The validity and/or reliability of screeners were investigated in 11 studies; responsiveness was not tested for any screener and practicality was considered in all studies. Based on the screeners reviewed, a systematic rationale to develop, enhance and test screeners based on national FBDGs was established.

## 1. Introduction

**Figure 1:**
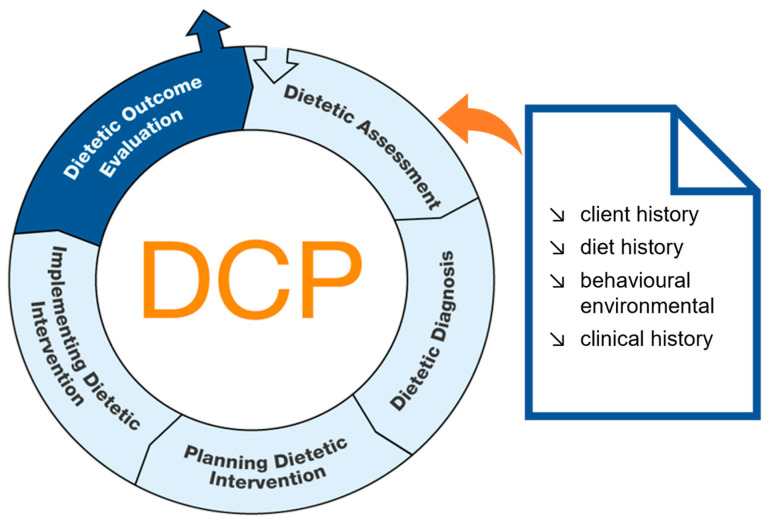
Dietetic assessment in the Dietetic Care Process (DCP) (adapted from [12,13]); the term dietetic assessment used in the DCP is synonymous with the term nutritional assessment.

Healthcare systems in the 21st century face an increasing burden from non-communicable diseases: in high-income countries, NCDs accounted for 87.8% of deaths in 2019 [1]. Given that non-communicable diseases are often modifiable through lifestyle changes [2], individualised dietetic counselling and therapy (DCT) is a key to reduce non-communicable-disease-related healthcare burdens [3,4]. Process models as a systematic approach for the whole dietetic intervention process, starting with an assessment and ending with an outcome evaluation, are needed for DCT. They ensure a high-quality DCT, e.g., by giving a common framework for DCT, support-evidence-based dietetic practice and a consistent documentation [5,6]. The nutritional assessment is the first step of process-guided DCT, which aims to collect high-quality data, particularly for determining dietary habits, in the so-called “diet history” (Figure 1). The diet history focuses on data on the usual intake of foods, energy and nutrients collected using different assessment methods and forms of administration, e.g., paper-based or digital, self-administered or interview-administered without a standardised approach [7]. Therefore, to ensure data quality, use of appropriate, evidence-based dietary assessment methods is required [4,6,8]. In addition, rapid assessment in DCT is important due to personnel and time restrictions, as well as client burden [9]. Currently, the majority of dietitians use their own assessment questionnaires for diet history, but desire standard tools [10]; therefore, the development of evidence-based, standardised and low-burden instruments is necessary [11].

**Figure 2:**
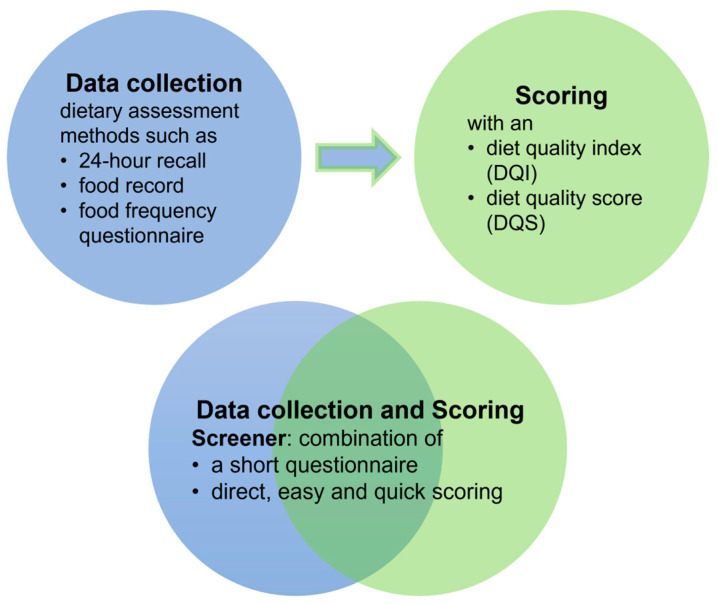
Approaches for determining diet quality (according to [11,16,17,18,20,28,29,30,31,32,33]).

The prevention and treatment of non-communicable diseases focus on healthy dietary patterns containing a variety of foods and food groups that are associated with diet quality instead of single nutrients [14,15,16,17,18,19]. In addition to common healthy dietary patterns such as the Mediterranean Diet and the Dietary Approaches to Stop Hypertension Diet [20,21,22], national, culture-specific, food-based dietary guidelines (FBDGs) have been developed in many countries (as of 2018, in 42% of countries worldwide) [23,24]. These guidelines focus on the prevention of non-communicable diseases and are also used in their management [25,26]. To determine individual diet quality, i.e., compliance with predefined healthy dietary patterns such as national FBDGs, a priori approaches are appropriate in both nutritional epidemiology and DCT [16,17,25,27]. A priori instruments summarise predefined dietary patterns into an overall measure of diet quality, usually using diet quality indices (DQIs) or diet quality scores (DQSs) [16,17,18,20,28,29]. Beyond DQIs and DQSs, which score previously collected dietary intake data, short instruments, so-called screeners or short dietary assessment instruments, have been developed. A diet quality screener is a short instrument that focuses on central diet quality aspects framed by predefined dietary patterns. It combines data collection as well as scoring, and is therefore ideal for use in evidence-based DCT where time and personnel resources are low [11,28,30,31,32,33] (Figure 2).

The consistent use of a screener to assess client diet quality could enhance the quality of food-related data collected as part of the diet history. This review aimed to give a comparative overview of screeners based on national FBDGs and to derive recommendations for developing a screener based on FBDGs for DCT. Therefore, the screener design should be considered, e.g., the theoretical framework or the indicator structure. Also, it should be considered if a newly developed screener was tested regarding common measurement properties such as validity or reliability. The review answers the following questions:

1. Which screeners are available that assess diet quality based on national FBDGs? How are the screeners designed and which measurement properties are tested?
2. What needs to be considered when developing a screener based on national FBDGs?

Details on the design and testing of screeners are presented and compared with general recommendations and specific advice for the design and testing of screeners in DCT. The review did not aim to recommend a single screener, but to examine the similarities and differences between screeners and support the development of national, FBDG-based screeners for DCT. Therefore, a full assessment of methodological quality and risk of bias for each study was not conducted.

## 2. Materials and Methods

This review was performed systematically, following the guidelines of the Preferred Reporting Items for Systematic Reviews and Meta-Analyses (PRISMA) [34,35].

### 2.1. Literature Search and Selection Process

For planning and conducting the review, the consensus-based standards for the selection of health measurement instruments (COSMIN) methodology for systematic reviews of Patient-Reported Outcome Measures was applied; this method can also be used for predictive or diagnostic outcome measures [36,37,38]. We determined the four COSMIN key elements (Table 1) to build a searchable question and derive search phrases (Table 2). The measurement properties were not included in the search strategy, in line with the COSMIN approach [36]. The whole planning process was performed by the first author (L.H.) and discussed with the last author (K.K.-M.). The literature search was conducted in PubMed (MEDLINE), embase and Web of Science and in the Register of Validated Short Dietary Assessment Instruments by the first author and discussed with the last author [39]. We used the COSMIN search filter for studies on measurement properties in PubMed (MEDLINE) and an adapted filter for embase [36]; in addition, the search was limited to adults. For Web of Science, no validated search filter exists, although a tool is under development [40]. For this reason, Web of Science was searched without a special search filter. Additionally, this database does not contain a filter for studies on adults; therefore, adult and grown-up were added as search terms. Finally, backward and forward hand searching was conducted. Studies that met the following criteria were included: full texts (post-prints) reporting on screener design and testing among healthy adults/adults with non-communicable diseases, including full screener information about design and testing. Only screeners that used FBDGs as a priori rationale and combined a simple questionnaire and scoring were included. If studies were available that presented items or scoring incompletely, we requested the missing information from the corresponding authors. We excluded articles about previous versions of further-developed screeners where the new instrument completely replaced the previous version, screeners on nutrient level intake or single food groups and screeners with complex scoring using extensive equations.

### 2.2. Data Analysis

**Figure 3:**
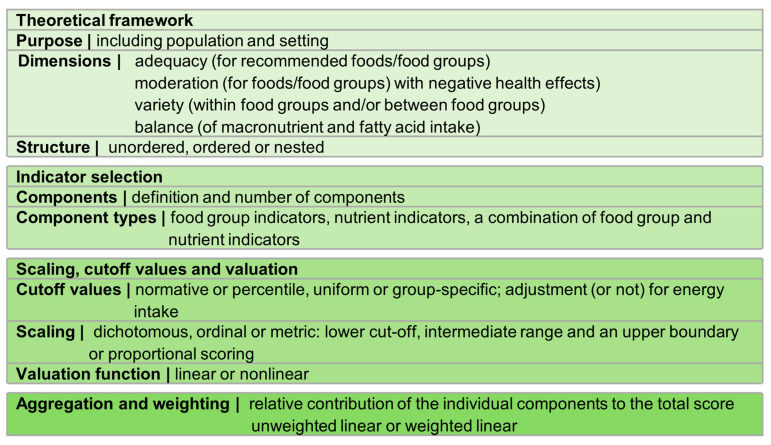
Characteristics included in the analysis of screener design (according to [11,16,17,33]).

Screener designs were analysed using criteria for screeners in DCT [11,33] and criteria for DQIs/DQSs [16,17]. Figure 3 presents the characteristics included in the analysis of the selected screeners.

**Figure 4:**
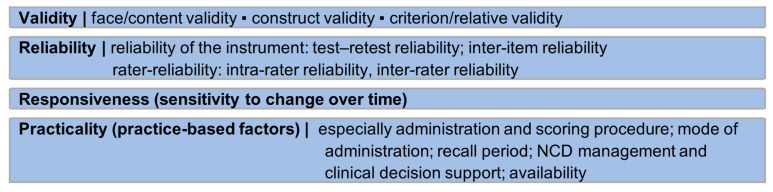
Measurement properties included in the analysis of screener testing (according to [11,33,36,41]). NCD: non-communicable disease.

Screener testing was analysed using measurement properties for screeners in DCT [11,33] and criteria for (dietary) assessment methods in general [36,41]. Figure 4 presents the measurement properties included in the analysis for screener testing [11,33,41].

## 3. Results

### 3.1. Study Selection

The initial literature search was conducted between May and July 2022 and updated in March and May 2023 by the first author (L.H.).

**Figure 5:**
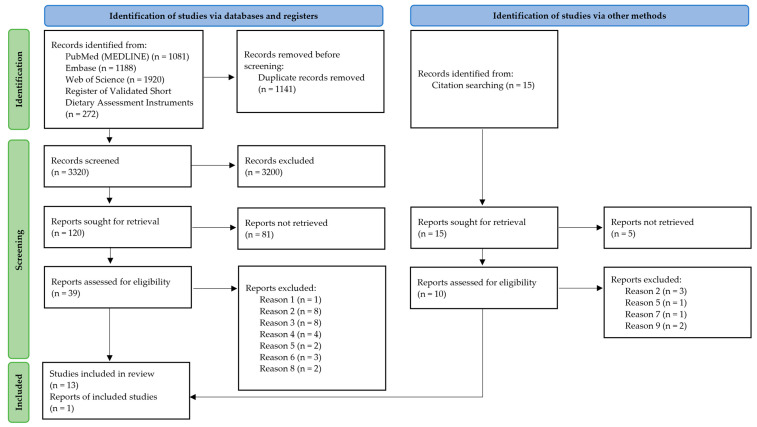
Article selection process represented using the PRISMA Flow Chart [34,35].

Of the 4461 studies identified via databases and registers, 3320 publications were screened after exclusion of duplicates using the literature management program Citavi (version 6.14). After screening, 120 articles were retrieved for review; of these, 39 publications were assessed for eligibility. Using a hand search, 15 studies were retrieved and 10 studies were assessed for eligibility. In total, 13 studies were included in the review through database, register and hand searches. In addition, one report containing additional data on a screener was requested from the authors. For the whole selection process, see Figure 5. Table 3 shows the exclusion reasons of the articles assessed for eligibility.

### 3.2. Characteristics of the Included Screeners

Thirteen studies of 11 screeners for assessing diet quality based on FBDGs were included in the review. Four screeners were developed in the USA [74,75,76,77,78], three in Australia [79,80,81,82], three in northern Europe [83,84,85] and one in Switzerland [86]. The studies on the Penn Healthy Diet Screener (PHDS) [74], Picture your Plate (PYP) [75] and the Score d’Alimentation Saine (SCASA) [86] were conducted between 2020 and 2022; the studies on the residential environments dietary guideline index (RDGI) [79], the Short Food Survey (SFS) [80], the Australian Recommended Food Score (ARSF) [81,82], the fifteen-item Food Frequency Questionnaire (15-item FFQ) [83] and the Food-Based Diet Quality Score (FBDQS) [84] were between 2015 and 2019; and the Index of Diet Quality (IDQ) was studied in 2010 [85]. The most recent study on the Rapid Eating and Activity Assessment for Patients (REAP) [77] was conducted in 2006; the first study on the Rapid Eating and Activity Assessment for Patients—Shortened Version (REAP-S) was in 2004 [78], with a further study in 2018 [76].

### 3.3. Screener Design

#### 3.3.1. Theoretical Framework

Purpose: Most screeners focused on the healthcare setting, i.e., DCT [74,75,76,77,78,83,86], and the IDQ was developed for epidemiological and clinical studies and DCT [85]. In contrast, the RDGI and SFS were developed for use at a population level [79,80], and no information on the purpose of use for the ARSF and the FBDQS was available [81,82,84].

Dimensions: All screeners used different dimensions for different food groups. For recommended food groups/items, they used the adequacy dimension; this included vegetables in all screeners and fruits in all screeners except the SCASA. For less recommendable food groups/items, i.e., those associated with adverse health effects, the screeners used the moderation dimension. This included, for example, processed meat products [75,76,77,78,79,86] and sugar-containing beverages [74,75,76,77,78,79]. PYP used a combination of adequacy and moderation for starchy vegetables and low-fat milk (product) items [75], the ARSF for different meat (products) [81,82] and the SCASA for fruits, oil-rich fruits and total consumption of protein-rich foods [86]. In terms of the variety dimension, the SFS used dedicated questions to record the variety of vegetables, fruits, grains, dairy products and protein-rich foods eaten [80], PYP assessed vegetable intake by listing individual vegetable subgroups (e.g., dark green vegetables, red and orange vegetables, starchy vegetables) [75] and the ARSF listed single foods in different food groups [81,82]. Hendrie et al. (2017) used food quality as a further dimension [80]. This dimension recorded specific characteristics of foods/food groups, especially within single food groups (e.g., whole grain/wholemeal bread, type of milk). In some other screeners, there were characteristics that could be included in this dimension, such as trimming the fat from meat [75,77,79] and the type of oils and fats consumed [75,80,83,85,86]; the PHDS termed questions of food quality as food behaviour questions [74]. No screener addressed the balance dimension and many food groups could not be assessed using a single dimension. Thus, in some screeners, items within food groups were assigned to different dimensions, e.g., for cereals, screeners used different dimensions: moderation for white cereals [74,75,84] and adequacy for whole grain cereals [74,75,76,77,78,84]; or adequacy and moderation for cereals and quality for whole grain cereals [79,80,81,82,83,85,86]; or variety of different cereals consumed in a day [80].

Structure: Except for the PHDS [74], screeners ordered their items by food groups [75,77,79,80,81,82,85]. The PHDS ordered their items by the different dimensions. The PHDS, REAP-S, 15-item FFQ, FBDQS and SCASA consisted of a total score calculated from the sum of single food items without subscores [74,76,78,83,84,86]. While scores for PYP, the REAP, ARSF, RDGI, SFS and IDQ comprised subscores representing different food groups [75,77,79,80,81,82,85], subscores for the SFS were according to food group and dimension [80]. In all screeners, a higher total score indicated higher diet quality. Four of the screeners classified diet quality based on the total score: PYP, the 15-item FFQ and the FBDQS described the classification a priori [75,83,84], while the IDQ classified diet quality based on data from the validation study using a receiver operating characteristic curve [85], analogous to percentile cutoffs as described by Burggraf et al. (2018) [17].

#### 3.3.2. Indicator Selection

Components: The number of items included differed between the screeners. Eight screeners included 35 items or less [74,76,77,78,79,83,84,85,86], the ARSF contained the highest number of items (*n* = 70) [81,82]. In addition to differences in food group intake, some screeners collected information associated with the diet history but beyond food-related recommendations; aspects related to out-of-home consumption [75,76,77,78] and meal frequency [76,77,78,83,85]; as well as behavioural environmental information (physical activity [77]) and clinical history details (body weight [86]).

Component types: In line with the inclusion criteria, all screeners were based on FBDGs and assessed food (group) intakes, not nutrient intakes. The PHDS included the Healthy Eating Index (HEI)-2015 and considered the Alternative Mediterranean Diet, the Dietary Approaches to Stop Hypertension Diet and the 2020 American Heart Association (AHA) Diet Goals [74]. PYP was a further development of a screener that focused on the intake of individual nutrients and was based on FBDGs and Recommended Dietary Patterns to Achieve Adherence to the AHA/American College of Cardiology Guidelines (AHA Recommended Dietary Pattern) [75]. The REAP-S was developed from the REAP by shortening and partially updating the items [76,78]. The RDGI, SFS, ARSF, 15-item FFQ and SCASA took into account already existing (inter)national indices, indicators and/or food frequency questionnaires (FFQs) in addition to FBDGs [79,80,81,82,83,86]. The IDQ was based on FBDGs supplemented with current scientific findings [85] and the FBDQS was developed from the IDQ [84]. In the SCASA, the link between food groups and nutrients was clearly visible, e.g., by scoring milk products separately for protein and calcium [86].

#### 3.3.3. Scaling, Cutoff Values and Valuation

Scaling: Six screeners used ordinal-scaled items—the REAP, REAP-S and RDGI for all items [76,77,78,79], and the PHDS, PYP and the SCASA for most items, with a small number of dichotomous items [74,75,86]. The item scoring ranges differed between screeners. The ARSF, 15-item FFQ, FBDQS and IDQ used dichotomous scaling [81,82,83,84,85]. The SFS was the only screener that used predominantly continuous (proportional) scaling [80]. In the PHDS, the number of questionnaire items (*n* = 30) differed from the number of scoring items (*n* = 15), since only those items that correlated strongly or moderately with the HEI-2015 were included in the scoring [74]. In the REAP-S, REAP, 15-item FFQ, FBDQS, IDQ and SCASA, the response options differed from the scoring options: the screeners allowed a high number of response options that were reduced to a smaller number of scoring options [76,77,78,83,84,85,86].

Cutoff values: All screeners used cutoffs—the screeners with ordinal or continuous scaling setting a minimum and a maximum cutoff [74,75,76,77,78,79,80,86], as well as intermediate ranges for ordinal scoring [74,75,76,77,78,79,86]. All screeners used normative cutoffs, although these differed between screeners; e.g., for vegetables, the cutoffs for the maximum score ranged between a consumption of at least two portions a day [76,78] and at least six portions a day (for men between 19 and 70 years) [79]. For PYP, the REAP-S, REAP, ARSF and IDQ, the reference source for the cutoffs was not declared. The studies generally referred to the use of FBDGs as well as other scientific evidence (see indicator selection) [74,75,76,77,78,81,82,85]. The RDGI, SFS, 15-item FFQ, FBDQS and SCASA used national FBDGs, the SCASA included additional scientific evidence and the FBDQS applied the IDQ approach [79,80,83,84,86].

Energy intake: Most of the screeners considered energy intake indirectly through quantity cutoffs—six screeners used portion sizes in household measures (e.g., hand, cup, glass, slice, piece) or metric measures (g, mL, oz) for (almost) all quantity items [75,76,77,78,79,85,86] and four screeners for only a few quantity items [80,81,82,83,84]. In terms of portion sizes, PYP, the ARSF, FBDQS and SCASA gave a selection of portion sizes within the response options [75,81,82,84,86]; the RDGI, SFS, 15-item FFQ and IDQ allowed open entry of portion size [79,80,83,85]; and the REAP-S and REAP included portion sizes in the questions [76,77,78]. Gender-specific portion sizes were included in the SFS [80], PYP and the REAP for alcohol [75,77], and in the RDGI for vegetables, milk and beverages [79]. Age-specific portion sizes were included in the RDGI for vegetables, milk and beverages [79].

Valuation function: Most of the screeners used linear valuation functions for their items. Valuation in some screeners increased up to the maximum recommended consumption and decreased with overconsumption for certain foods: low-fat/non-fat dairy items in PYP, meat product items in the ARSF, bread consumption in the 15-item FFQ and fruit and protein-rich food consumption in the SCASA [75,81,82,83,86].

#### 3.3.4. Aggregation and Weighting

In the REAP-S, 15-item FFQ and FBDQS, individual items were not weighted due to an unnested screener structure [76,78,83,84]. In the PHDS, the ordinal-scaled questions for adequacy and moderation ranged between 0 and 5 points and the dichotomous-scaled items for food behaviour ranged between 0 and 1 point [74]. The SCASA assigned a different scoring for vegetable consumption (−1 to 2 points) and for exceeding normal weight (−2 to 1 points) compared to for all other items (−1 to 1 points) [86]. No explicit weighting could be identified for PYP, the REAP, ARSF and IDQ [75,77,81,82,85]. Due to different numbers of items within subscores, the maximum possible subscore for each food group varied between screeners; therefore, the contribution of different food groups to the total score was inconsistent [75,77,81,82,85]. In the RDGI, each subscore could be a maximum of 10 points, although each subscore group contained a different number of items [79]. The SFS scored food groups without scoring individual items and maximum subscores differed between food groups [80].

### 3.4. Measurement Properties

Screener measurement properties were based on the criteria in Figure 4 (validity, reliability, responsiveness, practicality) and are presented in Table 4. Where available, the type of validity/reliability measured as well as the reference method (criterion/relative validity), time between measurements (test–retest reliability) and study design were identified.

#### 3.4.1. Validity and Reliability

In four of the eleven studies that assessed validity and/or reliability, the authors did not (completely) use the classification of validity/reliability in the forms shown in Figure 4, but addressed validity/reliability in general [77,78,80,85]. For this review, the type of validity/reliability measured was added by the authors.

The REAP-S was tested for relative validity by Segal-Isaacson et al. (2004), the ARSF was tested by Ashton et al. (2017) [78,81] and the IDQ was tested for relative validity, sensitivity and specificity [85]. PYP, the SFS and the ARSF by Collins at al. (2015) were tested for relative validity and test–retest reliability [75,80,82]; PYP was also tested for content validity, but this was not part of the publication [75]. The SCASA study assessed content and face validity, internal consistency, construct validity and inter-method reliability [86], while the PHDS was tested for content validity and compared with existing food recall data of the National Health and Nutrition Examination Survey (NHANES) 2017–2018 [74]. Masip et al. (2019) tested the construct validity of the FBDQS [84]. The 15-item FFQ was tested in a feasibility study where criterion validity, validity compared with health outcomes and cardiovascular risk factors were tested [83]. For the REAP, Gans et al. (2006) performed a multilevel evaluation: the items in the REAP were tested in a feasibility study, and then relative validity and cognitive assessment testing was performed. Based on the results, the REAP was revised and subsequently retested for relative validity and test–retest reliability [77].

Two studies did not perform conventional testing of measurement properties: Johnston et al. (2018) tested the association between REAP-S and the HEI-2010 [76]. Bivoltsis et al. (2019) developed the RDGI and tested several versions (a long and two short versions) against each other [79].

#### 3.4.2. Responsiveness

The responsiveness or sensitivity to change over time was not tested in any screener [74,75,76,77,78,79,80,81,82,83,84,85,86].

#### 3.4.3. Practicality

Except the REAP, which tested practicality within a feasibility study [77], no screener addressed practicality explicitly. However, information was given in all publications: administration and scoring procedures were simple in all screeners and possible without software [74,75,76,77,78,79,80,81,82,83,84,85,86]. For clarity of language for the clients, readability was considered in PYP, the REAP-S and the REAP [75,76,77,78], while the PHDS checked comprehension of items by patients [74]. Regarding the mode of administration, all eight screeners that specified the completion type were self-completable [74,75,76,77,78,79,81,82,85,86]; this was either explicitly stated by the authors or taken from the methods of testing the screeners. Four screeners gave a completion time: these differed slightly between screeners regardless of the number of items (4–20 min) [74,75,77,81,82]. The recall period was specified for six screeners with large differences: the 15-item FFQ measured habitual intake without a specified time period [83], the PHDS measured a single day (or 1 week in a modified version that has not been tested yet) [74], the REAP-S and the REAP referred to 1 week [76,77,78], the ARSF specified a period of 6 months [81,82] and the FBDQS referred to the past year [84]. Not all screeners were tested for use in DCT (see above); of the screeners tested for DCT, PYP, the REAP-S and the 15-item FFQ specified their usefulness for chronic disease management, PYP and the 15-item FFQ for cardiovascular disease management [75,83] and the REAP-S for prediabetes [78]. Clinical decision support was provided for PYP [75] and the REAP [77]. The 15-item FFQ, FBDQS and IDQ also ranked scores to support assessment of overall diet quality [83,84,85]. All screeners were fully available (as required using the inclusion criteria); however, the SFS lacked scoring information (time perspective) [80], the FBDQS did not have the exact item wording available [84] and the SCASA questionnaire and score were available only by request (see study selection).

## 4. Discussion

Screeners are simple and quick methods to collect evidence-based data for diet history in DCT. In the 1990s, the World Health Organization and The Food and Agriculture Organization advocated FBDGs for simple nutrition recommendations [87,88]. After the implementation of FBDGs, the first screeners were developed in the early 2000s, even specifically for use in client-centred DCT [77,78]. A standardised screener for diet quality in patients with non-communicable diseases could aid decision making in an extensive nutritional assessment and supporting data collection for dietetic diagnoses and monitoring and dietetic outcome evaluation. However, it is important to note that a screener only gives a brief overview of diet quality and is not based on meals, unlike the usual approaches of food records or 24 h recalls in DCT [7].

Screener results could also be used for individualised DCT, based on the client’s lowest-scored food groups, an approach used in several recent studies. Zenun-Franco et al. (2022) tested a web-based intervention (the eNutri App) among healthy adults and made dietary recommendations using the three lowest-rated food groups with a DQI (personalised approach) and compared this with general advice; they concluded that web-based personalised dietary advice was more motivating than general advice [89]. The Eetscore, an instrument based on a short FFQ (Eetscore FFQ) and a DQS (Dutch Healthy Diet 2015 index), provides personalised feedback based on clients’ diet quality [60,70]. Lamers et al. (2022) tested the Eetscore in clients with inflammatory bowel disease. The clients received personalised advice based on the Eetscore and their health-related quality of life and clinical disease activity were assessed, both of which improved significantly. The authors concluded that the Eetscore is useful for assessment and advice among patients with inflammatory bowel disease [60].

### 4.1. Screener Design

Publications included in this review were analysed using recommendations for screeners in DCT and the recommendations for DQIs/DQSs in general; this allowed detailed comparison of the design of these tools.

The theoretical framework and basis for indicator selection were similar between publications, since the main inclusion criterion was use of FBDGs as a reference. For a comprehensive overview, screeners that were not specifically developed for DCT were included. However, it became apparent that the underlying construct for all screeners was quite fluent between FBDGs and other evidence-based findings or disease-specific recommendations. This is in line with DQIs/DQSs, such as the Diet Score developed for Germany, which is based on the FBDGs of the German Nutrition Society and current evidence [73].

The different dimensions were not explicitly specified in many of the screeners analysed, but they were included in their questions and scores. Adequacy and moderation were frequently used, while variety was rarely assessed; PYP, the SCASA and the ARSF combined the moderation and adequacy dimensions for some items. Existing DQIs/DQSs took a similar approach: for food (groups) with positive and negative health effects depending on the level of intake, the score was reduced in the case of under- or overconsumption [73,90,91,92]. The balance dimension was not used in the screeners, presumably because it is nutrient-based and too complex for a short screener. Instead, the quality dimension introduced by Hendrie et al. (2017) seems more suitable for a screener at the food (group) level, including quality aspects not covered with the adequacy and/or moderation dimension. This dimension is—although not specified explicitly—also included in other screeners.

Regarding structure, most screeners were ordered by food groups with or without subscores, allowing a quick, food-group-based analysis in DCT [11,31]. In addition, PYP, the FBDQS, 15-item FFQ and IDQ classified the diet quality based on the total score; this may be useful for clinical decision support and the management of chronic diseases (see practicality). An a priori (normative) classification was used in PYP, the FBDQS and the 15-item FFQ, while a classification based on percentiles was implemented in the IDQ.

As well as screeners being based on national FBDGs, additional recommendations were sometimes included, especially when the screeners targeted specific diseases, such as the AHA recommendations included in PYP for cardiovascular disease management. All screeners assessed food (group) intake according to national FBDGs that focused on foods and food groups and not on nutrient intakes [20,21,22,23,93]. In line with Vadiveloo et al. (2020) who recommended no more than 35 items for a quick screener [11], the majority of screeners did not exceed this number.

Previously, Burggraf et al. (2018) recommended metric scaling in screeners [17]; however, the majority of those assessed here used an ordinal and/or dichotomous scale. Therefore, ordinal scaling allows accurate as well as easy and quick scoring [11,16]. The REAP, REAP-S, FBDQS, 15-item FFQ, IDQ and SCASA used a higher number of response options than scoring options, which may not allow simple scoring [11]. In contrast, a higher number of answer options or open entry of portion sizes may increase representativeness and therefore usability for clients. The normative screeners’ cutoffs differed from each other, likely due to differences in population-specific FBDGs [24]. If energy intake was considered, this was by portion size, similar to the semiquantitative approach used in full FFQs [94]. Burggraf et al. (2018) also recommended that for food (groups) with positive and negative health effects depending on the level of intake, the score should decrease with under- or overconsumption [17]; however, the valuation function of screeners was predominantly linear, possibly based on the FBDGs used for reference. In some FBDGs, e.g., the German FBDGs, lower scores are assigned when consumption of food (groups) with a moderate recommended intake is either below or above the defined portion size (a combination of adequacy and moderation dimensions) [95]; meanwhile, the Australian Dietary Guidelines recommended a minimum recommended intake from several food groups, and the Dietary Guidelines for Americans tool MyPlate gave exact portion sizes for fruits, vegetables, grains, protein foods and dairy [96,97].

Finally, there was little information regarding aggregation and weighting in the screeners; thus, unintentional weighting may have occurred, for example, where different numbers of items were included in subscores.

### 4.2. Measurement Properties

Most screeners were tested for common measurement properties. With regard to validity and reliability, Vadiveloo et al. (2020) recommended testing criterion validity, which is specified in this publication as “criterion and relative validity”, taking into account general recommendations for assessment instruments [41], as well as test–retest validity [11]. Although most screeners were tested for criterion/relative validity and/or test–retest reliability, only a few papers differentiated between relative and criterion validity or specified test–retest validity. According to Kirkpatrick et al. (2019) differentiation between criterion and relative validity highlights the varying quality of reference instruments: for criterion validity, they recommend unbiased references, such as biomarkers, and for relative validity, error-prone reference instruments, such as traditional nutritional assessment methods (24 h recalls or food records) [41]. In addition, some of the studies included an additional analysis of the reference instrument at the food (group) level using DQIs to validate the screeners. Some studies used further forms of validity and reliability testing relevant to screeners: content and/or face validity, construct validity and inter-method reliability.

An assessment of responsiveness was also recommended by Vadiveloo et al. (2020) [11]. Although this was not tested in any of the screeners, this is consistent with best practice recommendations according to Kirkpatrick et al. (2019) who described the testing of responsiveness as challenging and rarely/never assessed for dietary assessment methods [41]. For DQIs, however, there are studies demonstrating the ability of DQIs to measure changes in diet quality; similar methods could therefore be used for assessing screener responsiveness [27].

Vadiveloo et al. (2019) and England et al. (2015) also recommended testing practicality [11,33]. Although practicality was not explicitly tested in any screener except the REAP, application of criteria from Vadiveloo et al. (2020) allowed information on practicality to be found in all publications. In the REAP study, different aspects of practicality were tested using a questionnaire within a feasibility study. Testing practicality might be possible with a pretest, as mentioned by Cade et al. (2002) for the development of FFQs [98]; depending on the target group, qualitative interview techniques could also be used [99].

### 4.3. Recommendations for the Design and Testing of a Screener Based on National FBDGs for DCT

**Figure 6:**
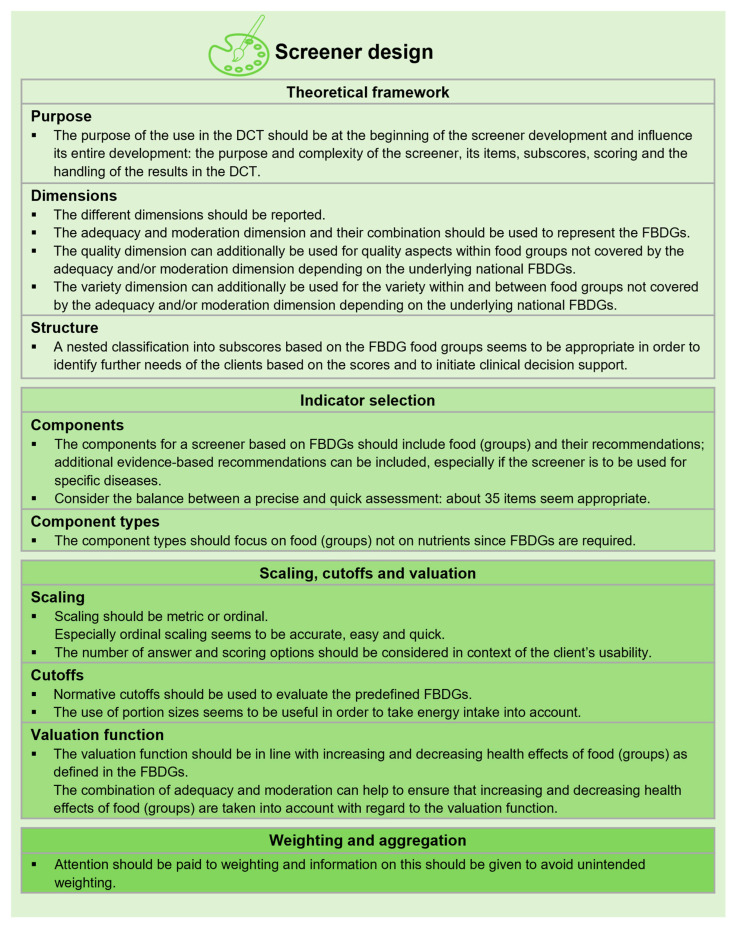
Recommendations for screener design. DCT: dietetic counselling and therapy.

**Figure 7:**
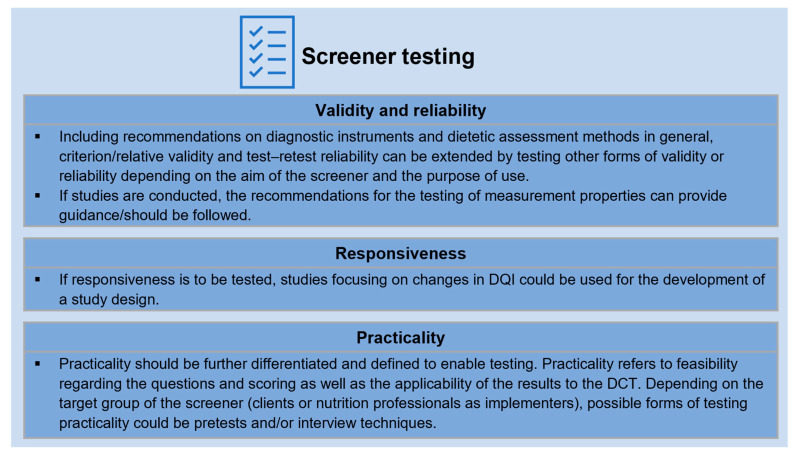
Recommendations for screener testing. DCT: dietetic counselling and therapy; DQI: diet quality index.

A systematic approach is necessary to design a screener with transparent data collection and scoring [11,16,17,33]; including detailed DQI/DQS characteristics (e.g., the theoretical framework, the indicator selection, scaling, cutoffs and valuation, as well as aggregation and weighting) is therefore helpful. According to evidence-based dietetic practice, a dietary assessment instrument needs to be tested for measurement properties; recommendations for screeners in DCT [11,33], as well as those for diagnostic instruments and dietary assessment instruments in general, could therefore be used [41]. Specifying the characteristics required for screener design, as well as the measurement properties and methods of testing, is key to establishing a robust screener. Differentiated recommendations for screener design and testing are given in Figure 6 and Figure 7.

### 4.4. Limitations

Firstly, for the literature search, different procedures were followed depending on the availability of search filters. This helped limit the high number of results in PubMed (MEDLINE) and embase, but was not required in Web of Science due to a missing published search filter and the lower number of matches. Secondly, we used COSMIN search filters; although these were developed for Patient-Reported Outcome Measures, the authors recommend them for any diagnostic tools [37,100]. Thirdly, the search and the selection of the studies were only carried out by one reviewer (L.H., first author); therefore, despite careful procedures, publication bias cannot be completely excluded. Fourthly, the inclusion and exclusion criteria led to the exclusion of studies where instruments were also called “screeners”, but they did not include both a questionnaire and scoring component, which could have excluded some otherwise relevant screeners. Screeners were included, however, even when the authors called it an “index” or “score”, if they included a questionnaire and score; the use of different terms demonstrates that there is still no generally used definition and classification of DQIs, DQSs and screeners. Finally, this review aimed to give details on the design and testing of screeners based on FBDGs and to derive recommendations for screener design and testing, but not to explicitly recommend one of the screeners researched. Therefore, the methodological quality and risk of bias in each study were not checked.

## 5. Conclusions

A priori-defined dietary scores, particularly screeners, provide a quick and easy way to identify individuals at an increased risk of “unhealthy” dietary patterns according to FBDGs. Screener use can contribute to quality assurance in DCT as follows:

Screener results can classify clients based on their diet quality and help decisions around whether a client needs extensive DCT.

Screeners can provide food (group) data useful for a dietetic diagnosis, especially for defining a dietetic problem.

Screeners can provide baseline data for the nutritional assessment, progress data for monitoring and outcome data for outcome evaluation.

However, screeners based on FBDG need to be country-specific. While FBDGs currently exist for 42% of countries, the screeners reviewed here only covered six; therefore, the development of more screeners is needed. The review offers a systematically established rationale to develop or enhance screeners based on national FBDGs.

## Figures and Tables

**Table 1:** Key elements of the review aim used to build the search strategy, as defined with COSMIN guidelines [37].

Construct	Population	Type of Instrument(s)	Measurement Properties
Diet quality based on FBDGs	Non-pregnant and non-breastfeeding adults 18–65 years old	Screener	Validity, reliability, responsiveness, practicality

FBDGs: food-based dietary guidelines.

**Table 2:** Search terms derived from key elements of the review aim (presentation based on COSMIN [37]).

Type of Instrument					Construct			
	index OR indices OR indicator * OR score * OR assessment OR tool * OR nutrition assessment ^M^; nutritional assessment ^E^	AND	short OR brief OR rapid	AND		Diet * OR nutrition * OR food *	AND	quality OR guideline *
OR	Screener *				OR	diet, healthy ^M^; healthy diet ^E^		

^E^ Emtree in embase; ^M^ MeSH term in PubMed (MEDLINE); * wildcard.

**Table 3:** Reasons for report exclusion after eligibility assessment.

Number	Reason	Reference Number
Reason 1	Studies whose instruments have been replaced with a newer version	[42]
Reason 2	Studies that do not define diet quality using FBDGs	[43,44,45,46,47,48,49,50,51,52,53]
Reason 3	Screener application (not development)	[54,55,56,57,58,59,60,61]
Reason 4	Questionnaire without directly related scoring	[62,63,64,65]
Reason 5	Evaluation at a nutrient level, rather than food level	[66,67,68]
Reason 6	Reviews, not individual screeners	[11,30,31]
Reason 7	Focus on single food groups	[69]
Reason 8	Questionnaire and/or scoring not available	[70,71]
Reason 9	Scoring without questionnaire	[72,73]

FBDGs: food-based dietary guidelines.

**Table 4:** Characteristics of screener testing in included studies.

Screener, Country, Reference	Referred Guidelines and Standards	Validity and Reliability		Practicality				
		Study Type and Approach; If Available: (1) Measurement Properties; (2) Reference Methods; (3) Time between Data Collections; (4) Study Design	Participants	Administration and Scoring Procedure	Mode of Administration (Time Required; Mode)	Recall Period	NCD Management, Clinical Decision Support	Availability
PHDS, USA, [74]	2015–2020 Dietary Guidelines for Americans (HEI-2015); Alternative Mediterranean Diet; Dietary Approaches to Stop Hypertension Diet; 2020 AHA Diet Goals	Assessment of screener item comprehension prior to testing	*n* = 4 expert clinical dietitians, *n* = 7 student participants, *n* = 10 patients	Easy, no software needed	4 min; self-completable	1 day	n.s.	Completely available
		Content validity: CVI	CVI: *n* = 11 expert dietitians; after screener revision: *n* = 7 experts					
		Screener scoring algorithm: Comparison of simulated screener responses from adult NHANES 24 h recall data with HEI-2015 components computed from the recalls	NHANES (WWEIA) component: participants n.s.					
PYP, USA, [75]	2015–2020 Dietary Guidelines for Americans; AHA Recommended Dietary Pattern	(1) Content validity (data n.p.)	A team of dietitians specialised in cardiovascular dietetics, experts in nutrition research	Easy, no software needed	15–20 min; self-completable (readability was checked)	n.s.	Rehabilitation of cardiovascular diseases; interim guidance for interpreting the score	Completely available
		(1) Relative validity; test–retest reliability; (2) semiquantitative Harvard/Willett FFQ (HWFFQ), AHEI, DASH score; (3) 1 week; (4) crossover design	IC: all English-speaking patients referred to cardiac rehabilitation; validity testing: *n* = 108, 66 ± 12 y, BMI of 30 ± 6.7 kg/m^2^, 68% male, 70% primary indication for CR including a recent ischaemia-related cardiac event; test–retest reliability testing: *n* = 94					
REAP-S, USA, [83]	2000 US Dietary Guidelines for Americans; Healthy People 2010 objectives; REAP	(1) Assessment of the relationship between REAP-S and HEI (concurrent criterion validity); (2) 1 × 24 h recall, HEI-2010; health outcomes; (4) secondary analysis, data from a cross-sectional study	*n* = 81 healthy vegetarian and omnivorous adults, *n* = 27 omnivore, *n* = 26 vegetarian, *n* = 28 vegan, age 30.9 (±8.5) y, 70% female, BMI of 22.8 (±2.8) kg/m^2^	Easy, no software needed	n.s.; self-completable (readability was checked)	1 week	n.s.	Completely available
REAP, USA, [84]	2000 US Dietary Guidelines for Americans; Healthy People 2010 objectives	Feasibility study: quantitative survey using scale questions	*n* = 61 medical students, practicing physicians	Easy, no software needed	ca. 10 min; self-completable (readability was considered)	1 week	‘Physician Key’ to aid physicians in diet assessment and counselling	Completely available
		(1) Relative validity; (2) 3-d FR, HEI; (4) crossover design	*n* = 41 s-year medical students					
		Cognitive assessment testing: interviews	*n* = 31 staff, students (varying socioeconomic background), age of 32 (20–61) y, 62% female, 50% people of colour, 96% > college education, 76% income < USD 59,000					
		Validity and reliability (modified REAP based on the first three studies): (1) relative validity, test–retest reliability; (2) FFQ (by Fred Hutchinson Cancer Research)	IC: CS, >18 y, able to speak and read English; *n* = 94, 57% women, mean age of 43.2 (SD: 12.5) y, 94% non-Hispanic white, 57% high school graduates, median income range of USD 51,000–60,000.					
REAP-S, USA, [85]	2000 US Dietary Guidelines for Americans; Healthy People 2010 objectives	(1) Relative validity; (2) Block 1998 FFQ; (4) crossover design	*n* = 110 medical students, mean age of 24.2 (SD: 3.8) y, mean BMI of 23.4 (SD: 5.0) kg/m^2^, 53% male, 65% white	Easy, no software needed	n.s.; self-completable (readability was considered)	1 week	Management of prediabetes	Completely available
RDGI, AUS, [86]	Australian Dietary Guidelines; existing scores	Comparison of three indices: RDGI, S-RDGI1 and S-RDGI2 (containing different numbers of items); secondary analysis, data from quasi-experimental, longitudinal study (evaluating the impact of “Liveable Neighbourhoods Community Design Guidelines” on participant health and behaviour); associations between participant characteristics and RDGI scores	*n* = 555, age of 47.9 (SD: 11.9) y, 61.8% female, 37.3% with BMI 18.5–29.9 kg/m^2^, 35.9% with BMI 25–29.9 kg/m^2^, 33.7% with secondary education or less, 38.8% trade/apprentice/certificate, 56.8% income > AUD 90,000	Easy, no software needed	n.s.; self-completable	n.s.	n.s.	Completely available
SFS, AUS, [80]	2013 Australian Dietary Guidelines; existing scores	(1) Relative validity; test–retest reliability; (2) 3 × 24 h recalls (one weekend, two weekdays; 3-pass method); (3) 2 weeks; (4) crossover design	IC: CS, 19–50 years, living in Australia, adequate written and spoken English knowledge, internet access, no conditions affecting dietary intake and no plans to initiate dietary changes within the next month; *n* = 61, age of 34.1 (24–44) y, 72% female, >50% resided in higher socioeconomic areas	Easy, no software needed	n.s.; n.s.	n.s.	n.s.	Available with missing information
ARFS, AUS, [82]	Australian Dietary Guidelines; AES FFQ	Relative validity; test–retest reliability; (2) AES-FFQ; (3) 5 months; (4) secondary analysis, data from a crossover design	*n* = 96 (baseline); *n* = 67 (follow up); 48 females, BMI of 23.5 (22–26) kg/m^2^, 77% certificate/degree/postgrad; 31 males, BMI of 25.7 (24–28) kg/m^2^, 75% certificate/degree/postgrad	Easy, no software needed	10 min; self-completable	6 months (basic FFQ)	n.s.	Completely available
ARFS, AUS, [81]	Australian Dietary Guidelines; AES FFQ	(1) Relative validity; (2) biomarker: plasma carotenoid concentrations; (4) secondary analysis, data from a crossover design	IC: subset of participants from a previous weight loss RCT, overweight/obese, age of 18–30 y; *n* = 99, age of 44.6 (SD: 9.9) y, 94.5% female, BMI of 31.8 (SD: 3.8) kg/m^2^	Easy, no software needed	10 min; self-completable	6 months (basic FFQ)	n.s.	Completely available
15-Item FFQ, SWE, [83]	Nutrition Recommendations 2012; national indicators	(1) Criterion validity; (2) health outcomes: cardiovascular risk factors; (4) crossover design	IC: random sample of every fifth man and woman born in 1963 and living in the city of Gothenburg; *n* = 521, 51% women, BMI: 26.2 (SD: 4.42) kg/m^2^, 49.7% with university/college education	Easy, no software needed	n.s.; n.s.	Habitual consumption	Management of cardiovascular diseases; overall score ranking	Completely available
FBDQS, FIN, [84]	Nordic Nutrition Recommendations 2012; Finnish Nutrition Recommendations 2014; IDQ	(1) Relative validity; (2) 3-d FR (completed 4 years before testing the screener); (4) crossover design	Sample derived from wave five of the population-based FinnTwin16 (FT16) cohort study; main FT16 sample *n* = 3592, 56% females, *n* = 1878 with tertiary education, participants with lower DQ: BMI of 25.4 (25.2–25.7) kg/m^2^, participants with higher DQ: 24.2 (24.0–24.4) kg/m^2^, subsample with food diaries: *n* = 249	Easy, no software needed	n.s.; n.s.	12 months	Overall score ranking	Available with missing information
IDQ, FIN, [85]	Nordic Nutrition Recommendations 2004; current scientific evidence	Pilot testing	*n* = 14 healthy adults	Easy, no software needed	n.s.; self-completable	n.s.	Overall score ranking	Completely available
		(1) Relative validity; (2) 7-d FR; (4) crossover design	IC: healthy Finnish adults, age of 20–64 y; *n* = 103, mean age of 32 y, 83% women, 48% students, 77% BMI < 25 kg/m^2^, 46% following special diet					
SCASA, CHE, [86]	Swiss Dietary Guidelines 2011; existing scores	Content and face validity using interviews	*n* = 4 experts, *n* = 15 lay volunteers (heterogeneous regarding age, gender, socioeconomic status, BMI; without nutritional knowledge)	Easy, no software needed	n.s.; self-completable	n.s.	n.s.	Completely available upon request
		Internal consistency by pretesting SCASA	*n* = 30 lay volunteers (second-year bachelor’s students at the Geneva School of Health Sciences)					
		Construct validity by evaluating the ability of SCASA to discriminate balanced from imbalanced meal plans	*n* = 6 weekly meal plans created by dietitians					
		(1) Inter-method reliability; (2) 5–7-d FR; (4) crossover design	*n* = 105 lay volunteers, age of 30 (SD: 13.7) y, 73% women					

AHA: American Heart Association; ARSF: Australian Recommended Food Score; BMI: body mass index; CS: convenience sample; CVI: content validity index; d: day; DC: dietetic counselling; DQ: diet quality; FBDQS: Food-Based Diet Quality Score; FFQ: food frequency questionnaire; h: hour; HEI: Healthy Eating Index; IC: inclusion criteria; FR: Food Record; IDQ: Index of Diet Quality; NDC: non-communicable diseases; n.p.: not presented; n.s.: not specified; n.t.: not tested; PHDS: Penn Healthy Diet Screener; PYP: Picture your Plate; RDGI: residential e·nvironments (RESIDE) dietary guideline index; REAP: Rapid Eating and Activity Assessment for Patients; REAP-S: Rapid Eating and Activity Assessment for Patients—Shortened Version; SCASA: Score d’Alimentation Saine; SFS: Short Food Survey; WWEIA: What We Eat In America dietary intake interview component of NHANES; y: years; 15-item FFQ: Fifteen-Item Food Frequency Questionnaire.

## Data Availability

No new data were created or analysed in this study. Data sharing is not applicable to this article.
